# Improvement of parents’ oral health knowledge by a school-based oral health promotion for parents of preschool children: a prospective observational study

**DOI:** 10.1186/s12903-023-03567-x

**Published:** 2023-11-20

**Authors:** Zhiyi Shan, Chongshan Liao, Jiajing Lu, Cindy Po Wan Yeung, Kar Yan Li, Min Gu, Chun Hung Chu, Yanqi Yang

**Affiliations:** 1 Faculty of Dentistry, The University of Hong Kong, Hong Kong SAR, China; 2 Department of Orthodontics, Shanghai Engineering Research Center of Tooth Restoration and Regeneration, Stomatological Hospital and Dental School of Tongji University, Shanghai, China; 3 Taizhou Polytechnic College, Jiangsu, China; 4 Paediatric Dentistry & Orthodontics, Faculty of Dentistry, The University of Hong Kong, 34 Hospital Road, Sai Ying Pun, Hong Kong Island, Hong Kong SAR, China

**Keywords:** Oral health promotion, Oral health knowledge, Parent, Early childhood caries, Preschool children

## Abstract

**Background:**

Parents of preschool children have inadequate oral health knowledge in Hong Kong. Parents play a critical role in preschool children’s dietary patterns and oral health behaviors. A school-based oral health promotion (OHP) for parents of preschoolers was developed and investigated.

**Objectives:**

The objective of this study was to evaluate effects of the school-based OHP for parents of preschool children on parents’ oral health knowledge and preschool children’s early childhood caries (ECC).

**Materials and methods:**

This was a quasi-experimental study. Parents of preschool children were divided into the intervention group (IG) and the control group (CG) according to their own selection. Parents in the IG participated in a structured school-based OHP workshop, while those in the CG did not attend the OHP workshop. Parents in both groups were invited to complete a questionnaire assessing their oral health knowledge before (T0), one month after (T1), and twelve months after (T2) the OHP workshop. Preschool children’s caries was examined via dmft score at T0 and T2.

**Results:**

Parents’ oral health knowledge was negatively correlated with preschool children’s dmft scores (R = -0.200, P < 0.001). Oral health knowledge was significantly improved in IG (P < 0.001) but not in CG (P = 0.392) at T1. Both groups experienced a significant improvement in oral health knowledge from T0 to T2 (P < 0.001). Parents’ oral health knowledge in the IG was significantly higher compared to the CG at T1 (P < 0.001), but difference in the scores at T2 between the two groups showed no significant difference (P = 0.727). No significant difference was found in changes in children’s dmft score from T0 to T2 between the IG and CG (p = 0.545).

**Conclusion:**

Preschool children’s high ECC is associated with the limited oral health knowledge of their parents. The school-based OHP workshop for parents increased parents’ oral health knowledge within one month. This positive effect was maintained for twelve months and can be extended to a larger scale in the school setting.

## Introduction

Early childhood caries (ECC), defined as the presence of decayed, missing, or filled tooth surfaces in children’s primary tooth, is a prevalent dental disease occurring in children younger than six years of age [1]. ECC can rapidly progress and generate many serious oral health problems including tooth pain, infection and abscesses, premature tooth loss, and malocclusion. Untreated ECC even results in children’s difficulty in chewing, poor nutrition, slow growth, unsatisfactory school performance, and reduced quality of life [2–4]. Considering the deleterious impacts and rapid development speed, ECC should be prevented as well as managed at an early stage. ECC is a multifactorial disease. Innate factors like enamel defect and high levels of *Streptococcus mutans* can raise the sensitivity of ECC establishment. Environmental factors such as children’s diet and oral hygiene practices play a vital role in ECC development. Moreover, sociodemographic factors including parents’ education level, socioeconomic status, and oral health knowledge were also reported to have a substantial influence on children’s ECC [5–8].

Hong Kong is a Special Administrative Region of China with one of the densest populations globally [9]. According to the region-wide survey in 2021, there were nearly 300 000 preschool children (aged 0 to 5 years old) in Hong Kong [10]. More than half of Hong Kong preschoolers have ECC experience and over 90% of the decayed teeth were untreated [11]. Currently, the Hong Kong government does not provide any stipend dental services targeting preschool children. Children can only access the “School Dental Care Service (SDCS)” when they enter primary schools [12]. Due to financial concerns, most parents have not taken their preschool children for regular dental checkups, and therefore, they are unlikely to obtain any oral health-related information [10]. However, the aggressive nature of ECC puts children at a high risk of developing into more serious oral health problems, which can cause significant suffering before they can receive treatment from SDCS. Often, more complicated treatment procedures are required, leading to greater discomfort for children and heavier economic burden for the society [13]. Therefore, there is an urgent need to establish an effective and efficient strategy for controlling ECC in Hong Kong preschool children.

Parents play a significant role in children’s oral health behaviors and habit formation [14–16]. Previous research indicated that children’s development of food preferences, energy intake, and eating behaviors were substantially related to their parents and the family environment [17, 18]. Additionally, parents can control children’s oral health-related practices such as sugar consumption and oral hygiene practices through restrictions, education, encouragement, and awards. Most parents guide children’s dental care based on their personal oral health attitudes and experiences [7]. Some parents misdiagnose caries in children as “tooth stains” which could be cleaned off, delaying prophylactic treatment until the children develop tooth pain [19]. Many parents mistakenly believe that if their child’s teeth do not hurt, then there are no oral health problems [20, 21]. As a result of this misconception and the rapid progression of ECC, dental care is often not sought until a child requires more invasive dental procedures including root canal treatment and tooth extraction. Therefore, improving parents’ knowledge of the importance of primary dental care is crucial for enhancing children’s oral health.

Oral health promotion (OHP) is a cost-effective strategy aimed at improving participants’ oral health knowledge, potentially increasing oral health awareness, and changing their oral health-related behaviors. The underlying mechanism for this theory is that recipients with increased oral health knowledge would alter their oral health behaviors. OHP programs implemented in kindergarten or school settings could reach the largest number of target children and can access their indispensable supporting networks [22]. However, school-based OHP programs focusing solely on preschool children can be quite challenging, as they need to take into account the children’s psychological and behavioral characteristics. More complex approaches, like incorporating games and drama, have been suggested instead of verbal instructions [23, 24]. Preschoolers are limited in their development stage and are not fully capable of understanding the long-term consequences of neglecting their oral hygiene or the significance of oral health. Furthermore, preschoolers do not have the autonomy to regulate their lifestyles including feeding patterns and hygiene practices. Considering the fact that parents play a pivotal role in children’s oral health, it is necessary that parents be the critical target for school-based OHP programs to improve their oral health knowledge, which may also contribute to controlling and managing children’s ECC.

Currently, there are limited studies exploring school-based OHP strategies to improve the oral health of preschool children. Even fewer studies have involved preschool children’s parents in OHP interventions. One recent systematic review identified only two studies (conducted in China and Argentina) that implemented OHP interventions for parents in the school setting; however, these interventions were also delivered to children and teachers [22]. While these comprehensive school-based OHP approach were effective in establishing better oral health habits, maintaining sound oral hygiene, and preventing the development of new caries and gingivitis, their time-consuming and labor-intensive nature may pose challenges, particularly when attempting to apply them to larger populations. To our knowledge, no efforts have been made to investigate the effects of the school-based OHP strategy specifically for parents of preschoolers on parents’ oral health knowledge and children’s ECC. In order to fill this research gap, we conducted a prospective observational study, aiming to evaluate the effects of the school-based OHP for parents of preschool children on parents’ oral health knowledge and children’s ECC in Hong Kong.

## Materials and methods

### Ethics approval

This prospective observational study was conducted in compliance with the Declaration of Helsinki and was approved by The University of Hong Kong and Hospital Authority Hong Kong West Cluster (HA HKW) Institutional Review Board (HKU/HA HKW IRB No UW12-334). All methods were carried out in accordance with relevant guidelines and regulations. Informed consents were obtained from a parent and/or legal guardian for study participation.

### Eligibility criteria of the study population

Ten kindergartens with preschool children aged 2 to 6 years old were selected using random stratified sampling based on the population distribution among the three territories of Hong Kong (Hong Kong Island, Kowloon, and the New Territories). The screening was conducted among child-parent pairs. The inclusion criteria for this study were: (1) Chinese ethnicity of both children and parents; (2) Good comprehension of Cantonese and traditional Chinese. The exclusion criteria were: (1) Children or patients with vision/hearing impairment; (2) Children or parents who had serious systemic diseases or mental health disorders requiring long-term medications; (3) Illiterate parents; (4) Children with severe craniofacial anomalies, syndromes, or cleft lip/palate.

### Sample size calculation

The oral health knowledge questionnaire for our study was specifically designed covering information of different aspects of risk factors and prevention of oral diseases in preschoolers. Due to the absence of studies using exactly the same assessment, the sample size of our prospective observational study was determined based on the previous study [25] investigating the effects of a 2-year oral health education program in Chinese kindergartens on “knowledge about toothpaste amount” to represent the general oral health knowledge. In order to detect a difference in the proportion of parents’ oral health knowledge between the test group (83.9%) and the control group (61.2%) with a significance level of 0.05 (two sides) and a power of 90%, a sample size of 174 was calculated using G*Power Version 3.1 software [26]. Taking into account a 50% attrition rate, at least 348 child-parent pairs should be included in the study.

### Study design and procedures

A general health screening form with demographic information required were sent to all parents of preschool children in ten kindergartens. For preschool children and their parents who met the eligibility criteria and agreed to participate were included in this study. Before the school-based OHP workshop (T0), parents’ oral health knowledge was assessed using a questionnaire in traditional Chinese which was specifically designed for parents of preschool children in Hong Kong. The questionnaire consisted of 47 multiple-choice questions covering information about the risk factors and prevention of oral diseases in preschool children. It was divided into four domains: recognition of proper oral hygiene behaviors, the influence of poor oral habits, factors relating to oral diseases (ECC, periodontal diseases, and malocclusion), and diet/snacking habits. The questionnaire for parents’ oral health knowledge was validated by five professorate staff in the Division of Pediatric Dentistry and Orthodontics in The University of Hong Kong. These professors were not involved in the designing process of this questionnaire. The questionnaire was also assessed for its internal consistency and test-retest reliability in the pilot test using Cronbach’s alpha and Intraclass Correlation Coefficient (ICC), respectively. During the pilot test, the questionnaire was distributed to ten parents of preschool children, and their responses to the items were analyzed using Cronbach’s alpha. After a two-week interval, the same ten parents were invited to complete the questionnaire again, and the ICC of parents’ oral health knowledge score was calculated. The results (𝛼 = 0.81, ICC = 0.86) indicated the questionnaire had good internal consistency and test-retest reliability. We present the English translation of the questionnaire in Table 1 to enhance understanding and communication. The same questionnaire was given to all parents after 1 month (T1) and 12 months (T2) of the OHP workshop. Parents received one point for each correct answer on the questionnaire, while a score of 0 was given for incorrect or “don’t know” answers. The total score for parents’ oral health knowledge could be ranged from 0 to 47.

**Table 1:** Questionnaire for the assessment of parents’ oral health knowledge

Oral health knowledge questionnaire		Yes	No	Don’t know
What cause(s) dental caries?	Hot air			
	Frequent eating			
	Frequent consumption of sweet food			
	Improper toothbrushing			
	Lack of calcium			
What prevent(s) caries?	Use of fluoridated toothpaste			
	Reduce consumption of sugar			
	Proper toothbrushing			
	Calcium supplement			
	Herbal tea			
What cause(s) periodontal disease?	Improper oral hygiene			
	Aging			
	Hot air			
	Forceful/ vigorous toothbrushing			
	Genetics			
What prevent(s) periodontal disease?	Proper oral hygiene			
	Rinse with salt water			
	Regular scaling			
	Herbal tea			
	Use floss or interdental brush			
What effect(s) does fluoride have?	No effect			
	To prevent periodontal disease			
	Tooth whitening			
	Prevent tooth decay			
	Flavoring in toothpaste			
When do the first permanent molars usually erupt? *	4 to 5 years old			
	5 to 6 years old			
	6 to 7 years old			
	7 to 8 years old			
	I do not know			
Which kind of teeth is the first type of permanent teeth to erupt? *	Incisors			
	Posterior molars			
	Others			
What cause(s) malalignment of teeth?	Bruxism			
	Tongue thrusting			
	Digit/ Thumb sucking			
	Genetics			
	Trauma			
	Mouth breathing			
	Severe caries			
Which of the following method is better to the oral condition? *	Breastfeeding			
	Milk bottle feeding			
What oral condition(s) would occur, if your child uses a milk bottle with milk or sugary drinks to sleep?	Jaw pain			
	Caries			
	Tooth malalignment			
	Bruxism			
What cause(s) erosion of teeth?	Fruit drinks			
	Acidic beverage			
	Sport drinks			
	Yogurt			
	Gastro-oesophageal reflux disease			
What cause(s) tooth wear?	Incorrect toothbrushing			
	Eating hard food			
	Bruxism			

* Please choose only one answer to the questionnaire item

Clinical examination was carried out among the included preschool children by one outreach dentist, who had been calibrated by a senior specialist in pediatric dentistry, using disposable dental mirrors and intra-oral light-emitting diode lights before OHP workshop (T0) and after 12 months of the workshop (T2). A diagnosis of dental caries was made according to the criteria recommended by the World Health Organization [27]. The dmft index was used to record the dental caries in the primary dentition of included children. A tooth will be marked as “decayed” when there is unmistakable cavitation on the occlusal, buccal, or lingual walls of the tooth, a detectable softened floor or wall, or filled tooth with signs of caries. The “missing” or “filled” tooth will be counted if it is due to caries. The same outreach dentist performed a repeated examination of 10% children subjects with a one-month interval for both assessments at T0 and T2. The reliability of the outreach dentist was calculated using the ICC as 0.96 by a statistician.

**Fig. 1:**
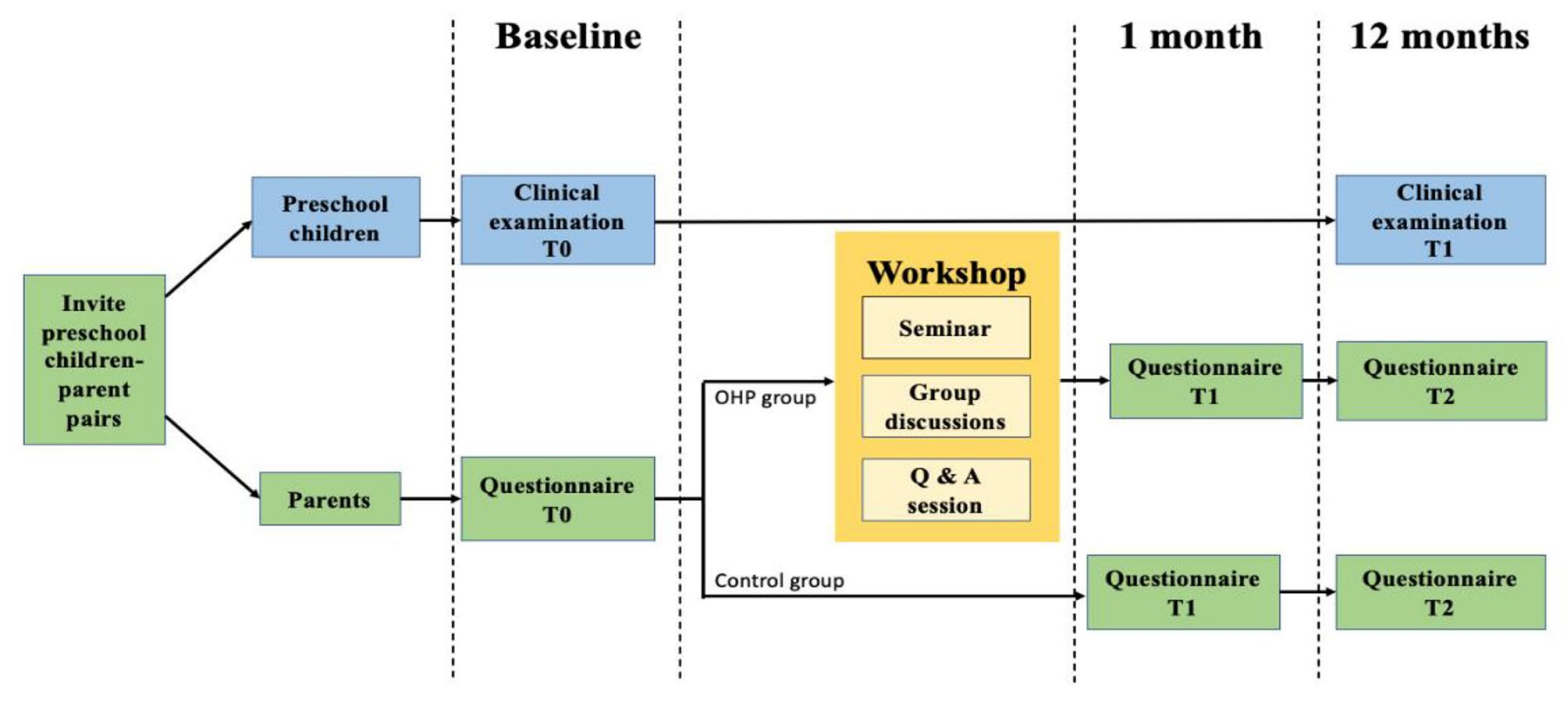
Flow chart of the quasi-experimental study design

All eligible parents were invited to attend a school-based OHP workshop delivered by a group of trained dentists. Those who were willing to come and managed to attend the workshop were designated to the intervention group (IG), while those who didn’t participate in the workshop were allocated to the control group (CG). The school-based OHP workshop for parents was designed based on the Health Belief Model (HBM) [28], which emphasizes the significance of parents’ perceptions of oral health in shaping their behavior towards oral health care for their children. These perceptions include the sensitivity and severity of their children’s oral health problems, as well as the benefits and barriers of children taking action to change oral health-related behavior. The school-based OHP workshop for parents consisted of a seminar, a series of group discussions, and a question-and-answer session. Parents were educated on the importance of maintaining good oral health, and the principles and practices of dental protection for guarding against the early signs of ECC were demonstrated. The workshop also covered the identification of malocclusion caused by poor oral habits and subsequent complications, along with protocols to break these habits (such as bottle feeding, thumb/digit sucking, and pacifier use). Oral health products and educational materials were also distributed among parents in the intervention group (IG). After 1 month (T1) and 12 months (T2) of participation in the OHP workshop, parents in both groups were invited to complete the same questionnaire to assess their oral health knowledge post-intervention. The flow path of the study design is illustrated in Fig. 1.

### Statistical analysis

Data analysis was performed using IBM SPSS Statistics for Windows (Version 28.0, IBM Corp., Armonk, NY). Demographic characteristics of parents were compared between IG and CG using chi-square tests. Total scores for parents’ oral health knowledge were presented as the mean ± standard deviation (SD) as descriptive results. The Pearson correlation coefficient was used to test the relationship between preschool children’s dmft score and parents’ oral health knowledge at the baseline (T0). Paired sample t-tests were used to compare the difference in parents’ oral health related knowledge between T0, T1 and T2. Independent sample t-tests were used to assess differences in parents’ oral health knowledge between the two groups. The significance level was set to 0.05, two sides.

## Results

**Fig. 2:**
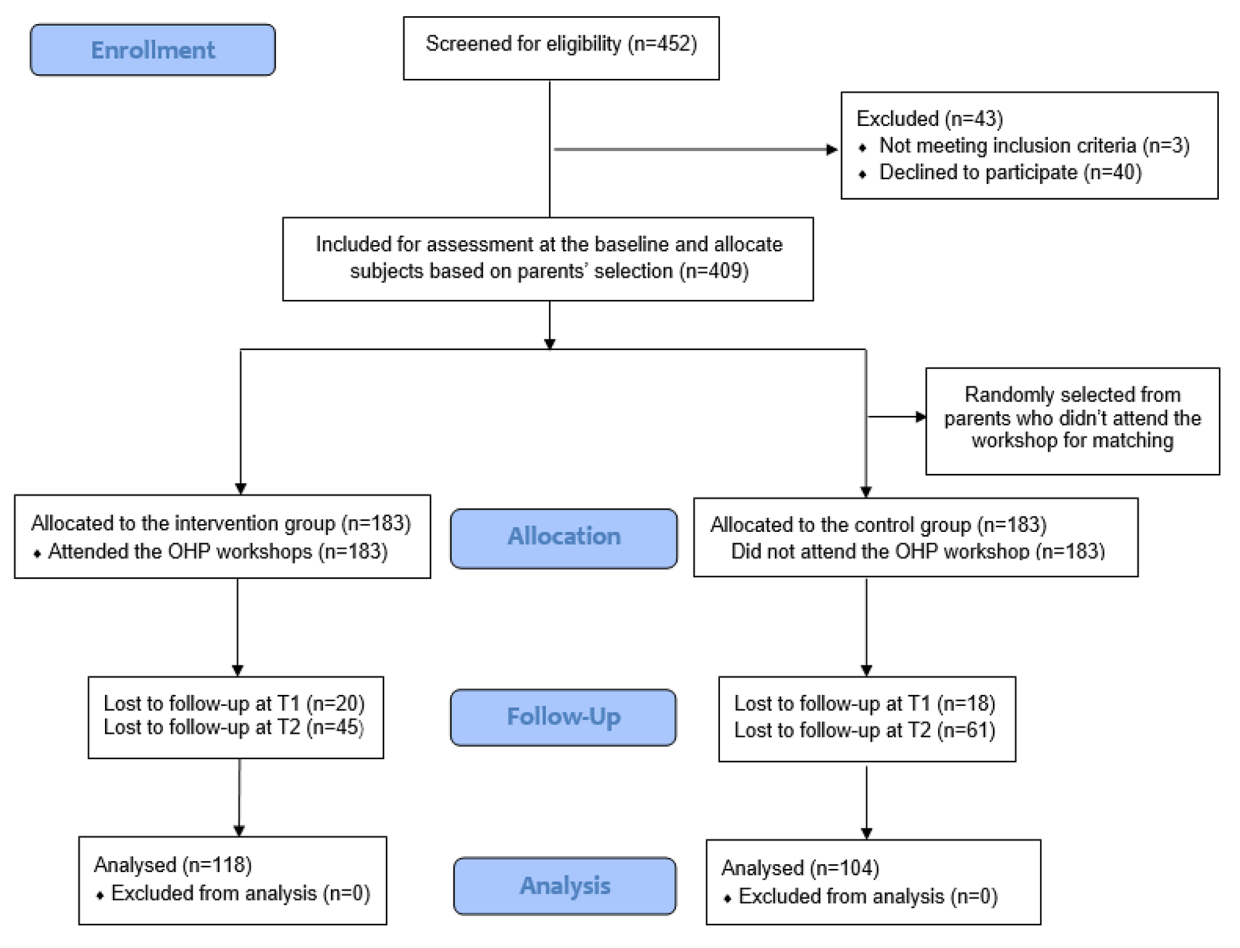
The flow diagram indicating recruitment, allocation, follow-up, and analysis of the study subjects

After screening subjects for eligibility, 409 pairs of preschool children and their parents were included in the study. Parents of preschool children who participated in the OHP workshop were allocated to IG (n = 183), and parents in CG were randomly picked from those who didn’t attend the workshop with the same number (n = 183) for matching purposes. Demographic analysis showed that there was no significant difference in parents’ age, education level, occupation, family income, and place of residence between the IG and CG (p > 0.05). In follow-up assessments, 39.3% of parents didn’t respond to the second and/or third questionnaires, as many children had either graduated or transferred to other kindergartens. In total, 222 parents (IG: n = 118 and CG: n = 104) completed the questionnaire at all three timepoints (T0, T1 and T2). Since the OHP workshop was non-harmful in nature, the missing data were assumed to be lost randomly and excluded from the analysis for parents’ oral health knowledge and preschool children’s ECC. The number of subjects for recruitment, allocation, follow-up, and analysis over the course of the study is depicted in Fig. 2.

### Association between parents’ oral health knowledge and preschool children’s ECC

**Fig. 3:**
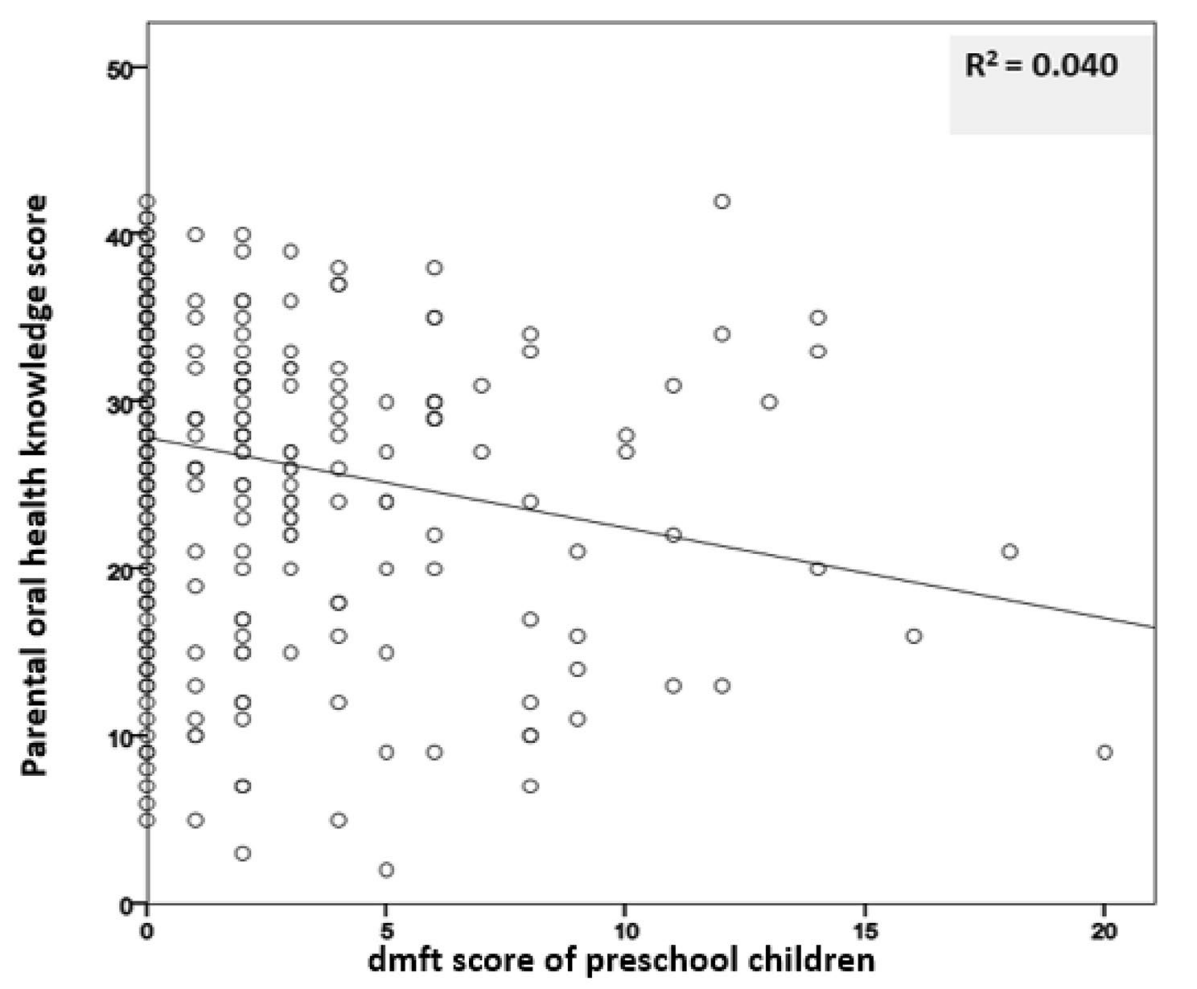
Relationship between children’s dmft score and parents’ oral health knowledge. The higher the score of parents, the lower the occurrence of caries of their children (R^2^ = 0.040)

On analyzing the full sample of child-parent pairs (n = 409) at the baseline (T0), results of the Pearson correlation coefficient analysis showed that there was a significant correlation between parents’ oral health knowledge and children’s caries experience assessed by the dmft score (P < 0.001). A negative correlation coefficient was detected (R = -0.200), which indicated that the reduction in parents’ oral health knowledge was associated with an increase in preschool children’s ECC (Fig. 3).

### Effects of the school-based OHP for parents on preschool children’s ECC

Results of independent sample t-test presented that there was no statistically significant difference in children’s dmft score (P = 0.162) between parents who attended OHP workshop (2.20 ± 3.77) and those who didn’t (1.68 ± 2.81) before the OHP workshop. After 12 months (T2), no significant changes were detected in children’s dmft scores for parents in IG (0.24 ± 1.66, P = 0.579) and in CG (0.10 ± 1.68, P = 0.147) from T0. Children’s dmft score change from T0 to T2 between the two groups was non significantly different (P = 0.545). (Table 2)

**Table 2:** Children’s dmft score in the intervention and the control groups before (T0) and 12 months after the school-based OHP (T2)

Early childhood caries (dmft score)	Intervention group (mean ± SD)	Control group (mean ± SD)	P-value
T0	2.20 ± 3.77	1.68 ± 2.81	0.162
T2	2.19 ± 3.53	1.62 ± 2.67	0.185
T2 – T1	0.24 ± 1.66	0.10 ± 1.68	0.545

### Effects of the school-based OHP on parents’ oral health knowledge

Result of the independent t-test showed that there was no significant difference in oral parents’ health-related knowledge before the OHP workshop (T0, P = 0.981) between the IG (26.79 ± 9.28) and CG (26.77 ± 8.73). One month after the OHP (T1), oral health knowledge of parents in the IG was significantly improved compared to the baseline (T1-T0 = 5.25 ± 8.59, P < 0.001), while that for parents in the CG did not present significant change over one month (T1-T0 = 0.61 ± 8.46, P = 0.329). One month after the OHP workshop, oral health knowledge between parents in the IG (32.04 ± 8.30) and the CG (27.38 ± 8.76) presented with a significant difference (T1, P < 0.001).

**Fig. 4:**
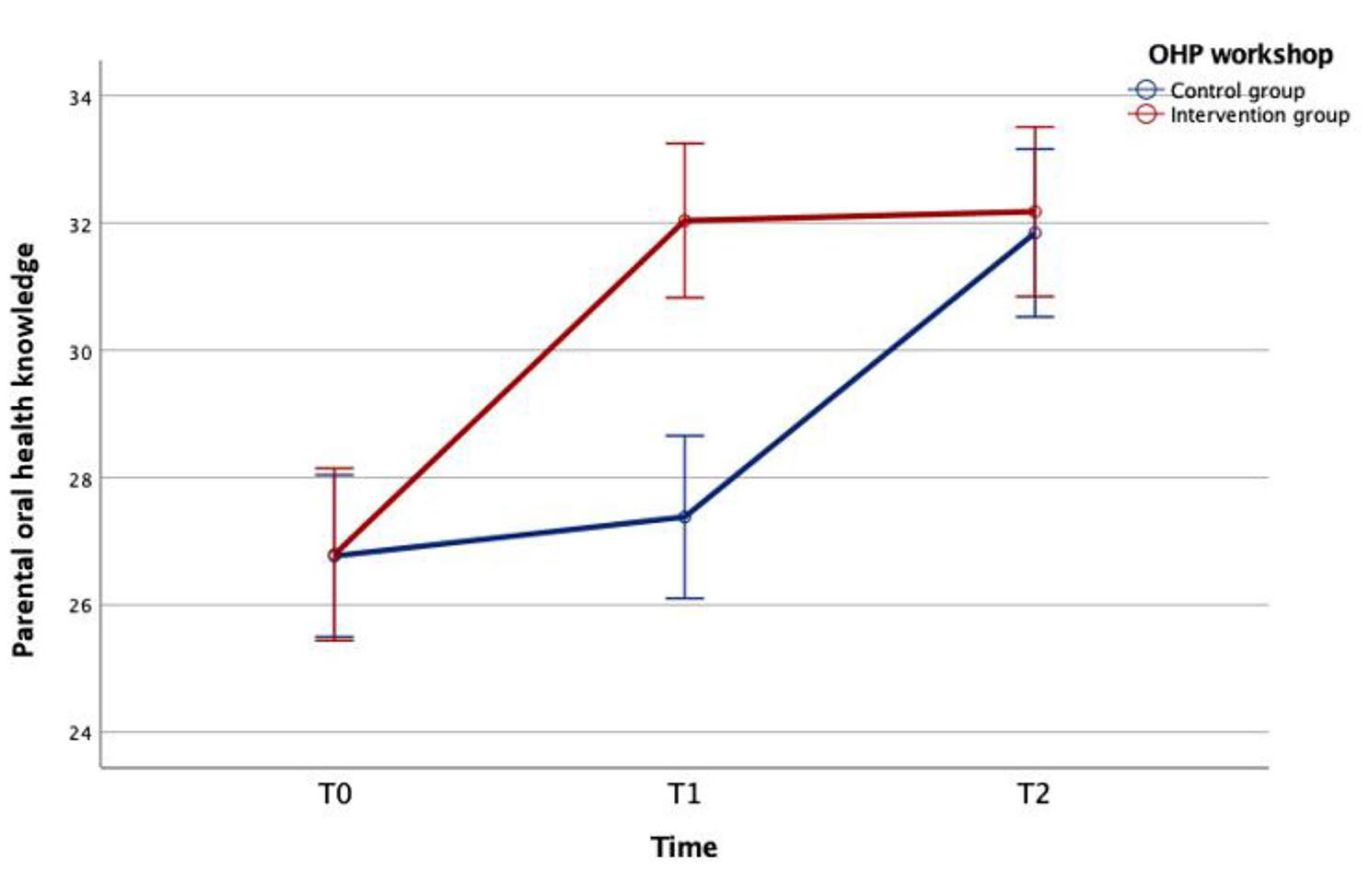
Changes in oral health knowledge of parents in IG and CG before (T0), 1 month after (T1), and 12 months after (T2) OHP workshop. The dental knowledge score of parents in the CG increased significantly from T0 to T2 and from T1 to T2, while the score of the IG increased significantly from T0 to T1 and from T0 to T2; there was a significant difference in the oral health knowledge of parents between the two groups at T1. (The results are shown as the mean and 95% confidence interval)

Twelve months after the OHP (T2), oral health knowledge of parents in both IG (32.18 ± 7.30) and the CG (31.85 ± 6.78) showed a statistically significant increase from the baseline level (T2-T0, P < 0.001). When compared to the T1 level, however, parents’ oral health knowledge was only significantly improved in the CG (T2-T1 = 4.63 ± 8.90, P < 0.001) but not in the IG (T2-T1 = 0.10 ± 7.42, P = 0.882). There was no significant difference in oral health knowledge between the IG and the CG at T2 (P = 0.727). Details of changes in oral health knowledge of parents in the two groups were shown in Fig. 4; Table 3.

**Table 3:** Parents’ oral health knowledge in the intervention and the control groups before (T0), 1 month after (T1), and 12 months (T2) after the school-based OHP workshop

Oral health knowledge	Intervention group (mean ± SD)	Control group (mean ± SD)	P-value
T0	26.79 ± 9.28	26.77 ± 8.73	0.981
T1	32.04 ± 8.30	27.38 ± 8.76	< 0.001
T2	32.18 ± 7.30	31.85 ± 6.78	0.727
T1 – T0	5.25 ± 8.59	0.61 ± 8.46	< 0.001
T2 – T1	0.10 ± 7.42	4.63 ± 8.90	< 0.001
T2 – T0	5.02 ± 8.83	5.00 ± 9.35	0.989

## Discussion

Oral health promotion programs aimed at preventative action are essential to help children to develop good habits of oral hygiene maintenance, promote their oral health, and improve their quality of life. Health habits and lifestyles established in childhood lead to positive outcomes that are lifelong. The oral health behavior of parents has a direct influence on their children [29]. Special attention should therefore be paid to the entire family in terms of their lifestyle and oral health habits.

This study found a negative correlation between children’s dmft score and their parents’ oral health knowledge. This confirms the importance of improving oral health knowledge among parents to benefit the oral health of preschool children. These results are consistent with previous studies. Crawford et al. [30] observed that poor parental oral health behavior was likely to be a predictor of caries in their children. Adair et al. [29] also demonstrated that children with satisfactory oral health usually have parents with decent oral health habits who monitor the children’s toothbrushing and daily sugar intake, suggesting that the oral health knowledge and attitude of parents have a positive effect on their children’s oral health status. Early oral health education and preventative measures help to reduce the need for future comprehensive treatment and even surgical intervention. However, it has been found that isolated OHP interventions without a supportive environment are not always successful in changing health behavior or achieving a sustainable health improvement [31, 32]. In this study, parents attended a school-based OHP workshop in consideration of the outcome of preschool children’s oral examination. In contrast to the traditional lecture format, the workshops comprised a seminar, group discussion and a question-and-answer session, aimed at helping parents assimilate the information, understand the instructions, and realize that they are important in the daily maintenance of their children’s oral health.

As the results demonstrated, parents’ oral health knowledge in the IG was significantly increased after 1 month of the OHP workshop, which was significantly higher than that for the CG. This indicated that OHP has a substantial effect on the improvement in the parents’ oral health literacy in short terms. However, it is also critical for any approach to children’s oral health achieving long-term effects [24]. To assess the sustained effectiveness of the OHP workshop, a 12-month evaluation was undertaken. Parents in the IG had a significantly higher oral health knowledge score at T2 than that at T0 (P < 0.001), indicating that the positive effect of OHP was maintained for at least 12 months. As for children’s caries development, we didn’t find a significant difference in the dmft score between the IG and CG groups one year after the OHP workshop. Also, there was no significant increase in children’s dmft score in both groups over the observation period. This might be associated with the influence of other OHP programs for preschool children in Hong Kong, including water fluoridation, oral health education among preschooler, and community dental services which parents and children can access voluntarily [33]. Additionally, although the Hong Kong government’s SDCS scheme has not yet covered preschool children due to possible financial constraints, inadequate infrastructure, and shortage of professionals, several projects conducted at the University of Hong Kong have provided outreach dental services with silver diamine fluoride to manage ECC since 2008 [34]. These OHP programs contributed to a lower dmft score for preschool children in Hong Kong compared to less developed regions including mainland China [35], India [36], and Thailand [37]. However, the dmft score is still higher relative to the Western countries [38, 39], highlighting the need for further exploration and development of effective OHP programs for preschool children.

In addition, we observed the oral health knowledge for parents in the CG, although did not attend the OHP workshop, was significantly improved after 12 months compared to the baseline (P < 0.001). This manifestation might be ascribed to the following three reasons. First, as all the kindergarten teachers attended the OHP workshops, they might have played a role in educating the preschool children and their parents over the 12-month period. Second, communication between parents may also have contributed to an improvement in the general level of parents’ oral health knowledge, meaning that the parents who had been educated in the OHP workshops performed as secondary educators and facilitated the spread of oral health knowledge. Third, all the parents received personalized reports following their children’s dental examination, and some followed the suggestions and sought dental clinics for further treatment. As parents receive individual oral health services during treatment, their knowledge of oral health may also increase.

The school-based OHP for parents not only benefits the oral health of preschool children but also provides personal benefits to parents by increasing their awareness of their own oral health status. This leads to greater oral health awareness for the entire family. However, the current attitude of the public in Hong Kong toward oral health is concerning. We sent invitations to all included parents (n = 409) and tried to increase participation by making phone calls and sending email reminders three days before the OHP workshops. Unfortunately, only 44.7% of parents attended. This reflect that some parents or families might not prioritize oral health high highly and may neglect existing dental services. Additionally, we noticed that many parents had a misconception that ECC in primary teeth did not affect the risk of tooth decay in permanent teeth, which may also prevent them from seeking necessary dental care for their children in preschool years. Future education efforts in Hong Kong should also focus on promoting a positive attitude toward oral health. The dental profession has a heavy responsibility in educating and promoting oral health among the public.

In summary, the findings of the study indicated a strong link between preschoolers’ dental caries experience and their caregiver’s oral health knowledge in Hong Kong’s population. The research suggests that a lack of parental awareness and understanding regarding oral health practices is significantly associated with an increase in ECC among preschoolers. The school-based OHP workshops proved to be an effective means of increasing parental understanding of oral health knowledge. After attending such workshops, parents’ oral health knowledge significantly improved after one month and twelve months. The study also underlined the necessity of improving parents’ oral health knowledge in kindergartens, especially in the Hong Kong region. Based on the study’s findings, efforts need to be made to enhance parents’ oral health knowledge through appropriate educational programs. The results of the study suggest that imparting knowledge and creating awareness among parents could decrease the likelihood of children’s ECC. It would help improve the oral health of future generations and lower the burden of public healthcare. Therefore, it is essential to design effective oral health education and awareness programs for parents in kindergartens to benefit preschoolers’ dental care. Governments and healthcare institutions could make continuous efforts to organize such programs to spread awareness regarding oral health among parents, leading to a healthier and happier generation.

However, this study has some limitations. First, due to ethical concerns, all eligible parents were invited to participate in the OHP workshop, and group allocation was based on parents’ preference rather than randomization. Parents’ awareness and attitude towards oral health in the two groups may have played a confounding role on parents’ oral health knowledge. Second, while the content of the questionnaire instrument for evaluating parents’ oral health knowledge had been validated by a group of experts, our study did not conduct other validity assessments such as construct validity and concurrent validity. This was due to the lack of consensus on oral health knowledge evaluation and limited studies specifically targeting parents of preschool children. It is important to make further efforts to develop a comprehensive instrument, both locally and globally, for assessing parents’ oral health knowledge of preschool children. Third, one-year observation was relatively short to detect significant changes in preschool children’s ECC. However, due to the constraints of the Hong Kong kindergarten settings and the high dropout rate of children every year after promotion or graduation, we only examined parents’ oral health knowledge and children’s ECC for one year. Future well-designed randomized controlled trials with longer follow-up period are still highly anticipated.

## Conclusions

This study found a negative correlation between preschool children’s ECC and their parents’ oral health knowledge in Hong Kong. The school-based OHP workshop for parents was an effective approach to increase parents’ oral health knowledge after one month. This favorable effect was able to last for twelve months and be transmitted to a larger scale in the school setting. Over the one-year observation, the school-based OHP workshop played no noticeable effects on children’s ECC. Future randomized controlled trials with long-term follow-up period are warranted to validate effects of the school-based OHP for parents on parents’ oral health knowledge and preschool children’s ECC.

## Data Availability

The data that support the findings of this study are available from the corresponding author, Y.Y., upon reasonable request.
